# A life-threatening complication of biliary peritonitis following T-tube removal: A case report and review of literature

**DOI:** 10.1016/j.amsu.2022.104209

**Published:** 2022-07-16

**Authors:** Yugant Khand, Sunil Basukala, Utsav Piya, Priya Mainali, Soumya Pahari, Kunda Bikram Shah

**Affiliations:** a Nepalese Army Institute of Health Sciences - College of Medicine, Sanobharyang, 44600, Kathmandu, Nepal; b Department of Surgery, Shree Birendra Hospital, Chhauni, Kathmandu, 44600, Nepal

**Keywords:** Choleperitoneum, T-tube, Biliary peritonitis, Case report

## Abstract

**Introduction and importance:**

The purpose of T-tubes is to induce inflammation around it in the common bile duct, forming a fibrous tract for drainage of bile. The leakage of bile into the peritoneum is a drastic complication following T-tube removal. A provisional diagnosis of choleperitoneum is established in the presence of persistent pain with guarding and rigidity. Imaging techniques can be used for the identification of biliary leakage. With most cases, patients recover with either conservative or surgical management.

**Case presentation:**

We present you a 65 years old malnourished female with features of choleperitoneum immediately following T-tube removal and was planned for conservative management with constant monitoring in surgical intensive care unit. The patient deteriorated despite adequate treatment and went into septic shock which resulted into her demise.

**Clinical discussion:**

Biliary peritonitis is not very uncommon but a life-threatening complication of T-tube removal. Poor nutritional status may also lead to delay in fistulous tract formation and there is a relative risk of biliary leakage during removal of T-tube. The use of a latex T-tube is more effective in mature tract formation and has less incidence of bile leakage. Seldinger's method, which involves using a wire to guide the removal of the T-tube, shows a significant reduction of biliary leakage.

**Conclusion:**

The mortality in biliary peritonitis significantly rises in cases of infected bile. The adverse reaction following the removal of T-tube was 4.3% and about 3% were severe enough to be admitted to the hospital.

## Highlights

• Biliary peritonitis is not very uncommon but a life-threatening complication of T-tube removal.
• Malnourishment causes delayed tract formation and increases risk of bile leakage during T-tube removal.
• Seldinger's method for T-tube removal shows a significant reduction in biliary leakage.

## 1 Introduction

External biliary decompression is achieved by placing a T-shaped tube into the common bile duct (CBD) following choledocholithiasis or repairing an iatrogenic CBD injury. The basis of T-tube placement is to allow the formation of fibrous bilio-cutenaous fistula in response to the inflammatory changes and prevent the leakage of bile into the peritoneum. The removal of T-tube is guided by the type of material used. In most cases T-tubes made up of polyvinyl chloride (PVC) and silicone are placed for 3 months whereas red rubber and latex can be removed after 7–10 days [1].

The only serious complication following T-tube removal is choleperitoneum which is a leakage of bile into the peritoneum leading to biliary peritonitis. The clinical features may depend on the amount of bile leaked into the peritoneum. In most cases, subclinical cases go unnoticed whereas patients may present with signs of peritoneal irritation. The prognosis of biliary peritonitis mainly depends on the extension of choleperitoneum. In most cases, the prospect of recovery is favorable with only a few mortalities occurring mainly due to infected bile.

There have been very few cases reported of biliary peritonitis following T-tube removal. We present here a potentially drastic complication of T-tube removal. This is reported in line with the SCARE criteria [2].

## 2 Methods

We reported this case following the updated consensus-based Surgical Case Report (SCARE) guidelines [2].

## 3 Case presentation

A 65 years old female with no known comorbidities presented with history of on and off pain in right upper quadrant (RUQ) for 6 months. The pain was acute in onset, pricking in nature, aggravated by oily food and there was no radiation to other sites. She complains of nausea and anorexia following the initiation of RUQ pain. She had no significant past and family history. She did not consume alcohol and was a nonsmoker. On clinical examination, she was afebrile without any signs of jaundice. The patient was underweighted with a body mass index (BMI) of 18.1 kg/m^2^ (height:156 cm, weight: 44 kg). The abdominal examination showed tenderness in RUQ with no features of rebound tenderness, guarding and rigidity. During her initial laboratory evaluation, complete blood count, serum amylase, serum lipase and liver function test were withing normal range. The patient was advised for ultrasonography (USG) of the abdomen and pelvis which showed dilated common bile duct (CBD) which measured 1.2 cm with multiple distal echogenicity causing posterior acoustic shadow. A provisional diagnosis of uncomplicated choledocholithiasis was established and further surgical management was planned.

The patient underwent open choledocholithotomy with CBD stenting and a T-tube was placed in the CBD. Intraoperative cholangiography was performed which confirmed absence of CBD stones. The patient was discharged on day 10 of admission with a healthy incisional wound, improving nutritional status (BMI: 20.3 kg/m^2^) and a T-tube in situ. She was advised to remove the T-tube after 2 weeks. She returned on day 30 following her operation for the removal of T-tube with a BMI of 16.8 kg/m^2^ (height: 156 cm, weight: 41 kg).

Following 60 minutes of removing the T-tube, the patient developed acute agonizing pain in the right upper quadrant with radiation to the right shoulder. The pain was associated with nausea and multiple episodes of vomiting. On examination, the patient was ill-looking with tenderness in the RUQ along with board-like rigidity and rebound tenderness. Her bowel sounds were intact. Her initial blood parameters showed raised white blood cell with increased neutrophils and a significantly low platelet count of 60,000 (Normal: 150000–450000/mm^3^). Contrast enhanced computed tomography (CECT) abdomen confirmed the presence of fluid in the peritoneal and pleural cavity. Biliary peritonitis was suspected clinically and she was planned for emergency diagnostic laparoscopy. However, her condition deteriorated quickly making her unfit for any surgical procedures. She was admitted to the surgical intensive care unit (SICU) after a quick sepsis related organ failure assessment (qSOFA) scoring of 3/3 for constant monitoring of her vital parameters and blood investigation as shown in Table 1.

**Table 1:** Laboratory parameters on admission and Day1 and Day4 of SICU.

S.N.	Laboratory test	Normal range	Admission	SICU D1	SICU D4
1.	Hemoglobin	13–17 g/dL	9.3	9.3	9.9
2.	White blood cell Neutrophils (N), Lymphocytes (L), Monocytes (M)	4–11 × 10^3/uL N: 40–80% L: 20–40% M: 2–10%	16.6 N:81 L:9 M:2	12.4 N:88 L:10 M2	11.8 N:86 L:9 M2
3.	Platelets	150000-450000/mm^3^	75	60	82
4.	Sodium	136-145 mEq/L	143	146	146
5.	Potassium	3.5–5.1 mEq/L	3.4	3.8	3.7
6.	Urea	13–43 mg/dL	68	78	48
7.	Creatinine	0.6–1.3 mg/dL	0.9	0.9	0.6
8.	PH	7.35–7.45	7.49	7.57	7.5
9.	PaO2	80–100 mm Hg	84.0	50	50
10.	PaCO2	35–45 mm Hg	43.7	39.1	38
11.	Bicarbonate	22-28 mEq/L	23	22	20
12.	Lactate	0.6–2.0 mmol/L	1.5	2.1	3.2

A course of broad-spectrum antibiotics was initiated along with intravenous fluids to manage her ongoing losses. The bile was positive for *Klebsiella pneumonia* and *Escherichia coli*. Antibiotic courses were adjusted according to the culture and sensitivity. She was managed with a team of gastro-surgeons, critical care physicians, anesthesiologist and nurses. Despite the multimodal management, the patient went into septic shock with continued fall in mean arterial pressure (MAP) and increase in lactate levels on third of SICU admission. She was managed with crystalloid infusion and vasopressors for the following day. On Day 4, her sepsis worsened into multiple organ dysfunction syndrome following which she had a cardiopulmonary arrest that led to her mortality.

The patient family member had signed a Do Not Resuscitate (DNR) request following her worsening conditions.

## 4 Clinical discussion

Biliary peritonitis is not very uncommon but a life-threatening complication of T-tube removal. There is a possibility of complication with premature T tube removal removal, with studies suggesting an incidence of 2.47% with removal in 21 days (Maghsoudi, 2005) and 19.6% with removal in 5–13 days (Domellöf, 1997) [3,4]. These incidences also project a declining trend in the occurrence of complication following T-tube removal in recent years. Return to the operation theater is seen in 0–4.3% of cases and mortality ranges from 0% to 1.8% [5]. Immunocompromised patients, such as those on steroid therapy and patients with poor nutritional status show a significantly increased risk of biliary peritonitis. Poor nutritional status may also lead to delay in fistulous tract formation and there is a relative risk of biliary leakage during removal of T-tube. This is a probable cause in our case which lead to leakage of biliary contents during T-tube removal and resulted in biliary peritonitis.

All cases of biliary peritonitis show symptoms of severe pain in the abdomen starting within 2 hours of T-tube removal. The site of pain is usually in the right hypochondrium region and the right shoulder. Most patients developed fever within 12 hours of T-tube removal and majority of them had multiple episodes of vomiting. The adverse reaction following the removal of T-tube was 4.3% and about 3% were severe enough to be admitted to the hospital [6].

The purpose of T-tubes is to induce inflammation around it in the common bile duct, forming a fibrous tract for drainage of bile. Latex tubes have been shown to encourage vigorous inflammation, whereas newer silicone-coated or polyvinylchloride (PVC) T-tubes show milder inflammatory reactions and unsatisfactory track formation. Excluding patients with latex allergies, the medical literature is in a consensus that the use of a latex T-tube is more effective in mature tract formation and has less incidence of bile leakage. The use of silicone coated T-tube in our patient and inadequate lag period before removal of T-tube might have been a probable cause for choleperitoneum. The severe undernutrition of our patient is a considerable factor to understand the additional time required for the maturation of tract from the usual 7–10 days.

The removal of T-tube is considered safe for around 7–10 days. However, common practice is to keep the T tube for a period of 21 days [7]. If silicon or PVC tubes are used, they are kept in place for longer periods. The placement of T-tube for longer duration has failed to show any benefits as the possibility of biliary leaks is similar to the standard removal time [8].

Alternative approach for T-tube placement is choledochorrhaphy following choledocholithotomy which is a primary closure of choledochotomy. This procedure avoids the insertions of the T-tube altogether. The insertion of T-tube was the obvious choice in the past as choledochorrhapy was associated with complications such as stricture formation and unsatisfactory common bile duct decompression. However, with advances in surgical techniques, recent studies show that choledochorraphy may have decreased the risk of complications compared to T-tube insertion. A study conducted by Lygisakis on 117 patients, suggests a decreased incidence of bacteremia morbidity and mortality with primary closure [9]. The study suggests T-tube drainage has a risk of exposure to microorganisms leading to infection. More recent studies show a decreased incidence of postoperative complications and decreased hospital stay in patients with primary closure (Zhang 2009, El-geidie 2010) [10,11]. A study also stated that biliary peritonitis is higher in the T-tube than in the primary closure group [12]. Keeping in view with the recent advances in surgical techniques and safer protocols, we can deduce that primary closure would have been an appropriate measure for patients following choledocholithotomy. However, a major advantage of the T-tube over primary closure is the ease of visualizing stones postoperatively by T-tube cholangiogram and therefore the procedure is still preferred amongst many surgeons.

Seldinger's method, which involves using a wire to guide the removal of the T-tube, shows a significant reduction of biliary leakage especially in cases of immunocompromised patients and cases of liver transplantation [13]. However, recent studies suggest that performing a fistulography to identify maturation of tract prior to the removal of T-tube especially in immunocompromised state has been effective in reducing the procedure related complications [14].

## 5 Conclusion

Choleperitoneum is not a very uncommon complication following T-tube removal. However, it can be associated with increased morbidity and mortality when it is accompanied with other comorbidities like malnourishment. The mortality significantly rises in cases of infected bile.

## Ethical approval

The case report is exempt from ethical approval in our institution.

## Source of funding

This research did not receive any specific grant from funding agencies in the public, commercial, or not-for-profit sectors.

## Author contribution

Yugant Khand (YK) = Conceptualization, Supervision, Yugant Khand (YK), Utsav Piya (UP), Priya Mainali (PM), Soumya Pahari (SP) = Writing - original draft, Sunil Basukala (SB), Kunda Bikram Shah (KBS) = Writing - review & editing.

## Research registration number

Not applicable.

## Guarantor

Sunil Basukala (SB), MD in Hospital Administration (MDHA), Master of Surgery (MS), Assistant Professor, Shree Birendra Hospital, Nepalese Army Institute of Health Sciences (NAIHS), 44600, Nepal. Email: sunil.basukala@naihs.edu.np.

## Consent

Written informed consent was obtained from the patient for publication of this case report and accompanying images. A copy of the written consent is available for review by the Editor-in-Chief of this journal on request.

## Provenance and peer review

Not commissioned, externally peer-reviewed.

## Declaration of competing interest

All authors declare that they have no conflict of interest.
